# Design of a novel wheeled tensegrity robot: a comparison of tensegrity concepts and a prototype for travelling air ducts

**DOI:** 10.1186/s40638-018-0084-8

**Published:** 2018-05-15

**Authors:** Francisco Carreño, Mark A. Post

**Affiliations:** Department of Design, Manufacture, and Engineering Management, University of Strathclyde, 75 Montrose Street, Glasgow, G1 1XJ UK

**Keywords:** Bio-inspired robots, Tensegrity, Wheeled mobility

## Abstract

Efforts in the research of tensegrity structures applied to mobile robots have recently been focused on a purely tensegrity solution to all design requirements. Locomotion systems based on tensegrity structures are currently slow and complex to control. Although wheeled locomotion provides better efficiency over distances there is no literature available on the value of wheeled methods with respect to tensegrity designs, nor on how to transition from a tensegrity structure to a fixed structure in mobile robotics. This paper is the first part of a larger study that aims to combine the flexibility, light weight, and strength of a tensegrity structure with the efficiency and simple control of a wheeled locomotion system. It focuses on comparing different types of tensegrity structure for applicability to a mobile robot, and experimentally finding an appropriate transitional region from a tensegrity structure to a conventional fixed structure on mobile robots. It applies this transitional structure to what is, to the authors’ knowledge, the design of the world’s first wheeled tensegrity robot that has been designed with the goal of traversing air ducts.

## Background

**Fig. 1:**
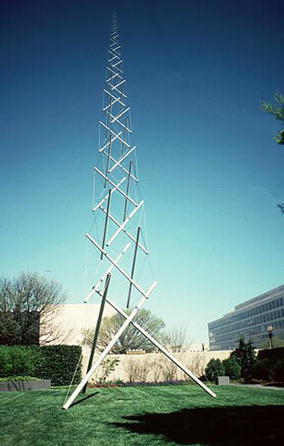
Needle Tower (© 2002 Mary Ann Sullivan). Located at Hirshhorn Museum and Sculpture Garden in Washington D.C., USA. Made by sculptor Kenneth Snelson. Spatial construction [1]. 1991 reconstruction of the 1921 original. Made by Latvian artist Karl Ioganson, it is the very first tensegrity structure ever made

**Fig. 2:**
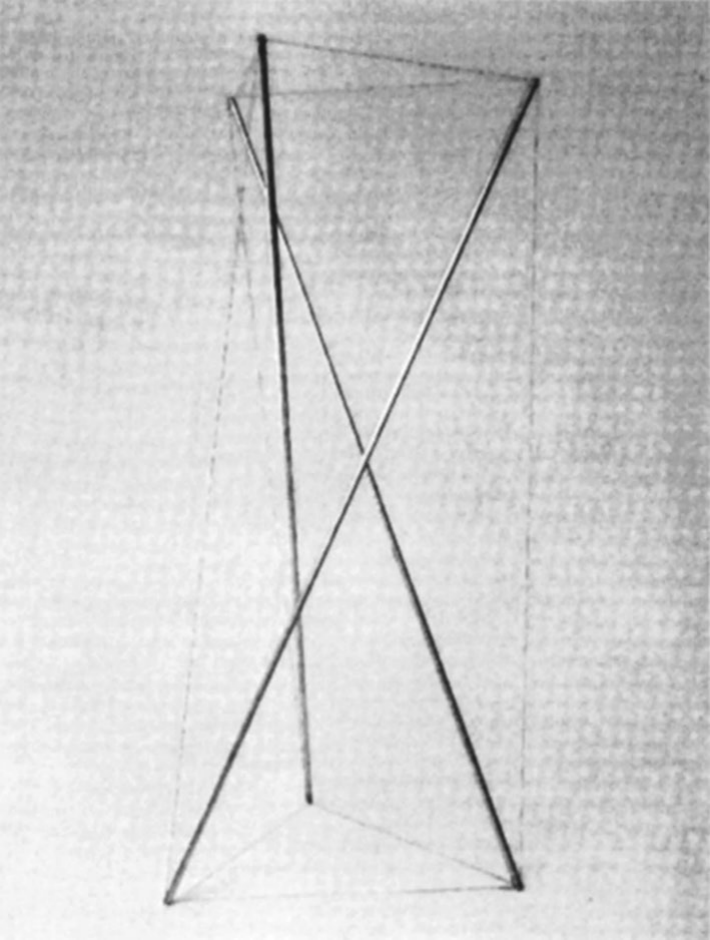
Spatial construction [1]. 1991 reconstruction of the 1921 original. Made by Latvian artist Karl Ioganson, it is the very first tensegrity structure ever made

A tensegrity structure is comprised of elastic elements and rigid elements that interact so the structure extends over a space larger than the space of its individual parts. The sum of the forces between all its parts is zero; therefore, the structure is in equilibrium without the need of external forces. All elastic elements bear exclusively tensional force, but not all tensional elements are elastic. The most spread-out use of rigid and elastic elements is struts and cables, respectively. The “Needle Tower” (Fig. 1) is made of aluminium struts and steel cables. The struts are suspended in the air held only by these cables. The structure rises with its internal energy compensating its potential energy. The first tensegrity structure was made by Latvian artist Karl Ioganson. It was shown in an exhibition in 1921 as part of his “Cold Structures” in the early Constructivism movement. Categorised at the time as a “Spatial Construction” (Fig. 2), the structure made is currently known as a 3-strut prism or triangular prism. Unfortunately, there was no further development of the novel structural principle [1]. Tensegrity structures were rediscovered by sculptor Kenneth Snelson who created “X Piece” (also known as “Early X piece”) in 1948 when he was still a student [2]. His professor, Buckminster Fuller, “asked Mr. Snelson to make a variation on “Early X Piece”, which he later exhibited—without crediting his student—at an important exhibition at the Museum of Modern Art in 1959” [3]. They both ended up filling patents to claim authorship in 1959 (patent number 3.063.521 granted on 1962 to Fuller) and 1960 (patent number 3.169.611 granted in 1965 to Snelson). There is also a patent (number 1.377.290) granted to Emmerich in France in 1963 [4]. The word tensegrity originated as the hyphenated word tensile-integrity structure, coined by Fuller in his patent. Similarly, Snelson coined the name continuous tension, discontinuous compression structures in his own patent.

Tensegrity structures eventually expanded beyond art into engineering. Many studies have been done in statics, kinematics, dynamics, and biology. The main fields focused in tensegrity are civil engineering and robotics. The latter have seen more than one thousand peer reviewed articles in the last 10 years. Research efforts in robotics span in all sub-categories of study inside tensegrity: form-finding, kinematics, dynamics, control, and locomotion. Form-finding is the search for new topologies and morphologies of tensegrity structures. It also explores new methodologies to be used for the search itself. One of the sub-areas of exploration is biotensegrity. This is the realm of tensegrity structures inspired by biology.

Most research papers focus on implementing tensegrity to address all design specification requirements. Tensegrity and biotensegrity structures are light weight, strong, flexible, compliant, and distribute the loads to every single element. Yet, when it comes to locomotive efficiency, these structures do not perform as well. A man with a bicycle is capable of travelling much faster and farther than a man running in spite of using the same source of power: the muscles in the legs. This work aims to join the advantages of tensegrity structures and wheeled locomotion. As a practical application of this new concept, it presents a biotensegrity structure that is able to climb through ducting by pressing wheels against the walls. The tensegrity structure is to provide the robot structural integrity as well as control of direction. The tensegrity structure, therefore, will need to provide enough flexibility to allow the robot to turn right, left, up, down, and in any other intermediate direction so the robot can change its path into other duct branches.

This paper is the first part of the above-mentioned study into wheeled tensegrity robotics. It focuses on the transitional region from tensegrity to a standard fixed mobile frame for wheeled locomotion. To the authors’ knowledge, there is no formal research work done on this area. It firstly introduces criteria of evaluation for a transitional region. Secondly, it reviews some of the available regular configurations of tensegrity structures as a preliminary study. Thirdly, it proposes new conceptual transitional regions to be validated later in the paper. Fourthly, it shows the design and prototype of a novel wheeled tensegrity robot. Finally, it presents discussion and conclusions. As a representative problem that can be solved by a tensegrity-based wheeled robot, we have chosen the mechanical traversal and navigation of ducting networks. Ducting networks in buildings and industrial facilities can span and branch out in any direction in space. Most mobile robots are designed to travel only in a horizontal plane and have difficulty turning in arbitrary directions in three dimensions. Robotic duct climbers are only able to travel in one line and maybe follow the duct mainstream through wide bends. They currently do not have the ability to turn out of the duct into a branch. A robot able to transit through air ducts in houses and buildings will reduce time and number of personnel required as well as increasing flexibility and scope of work done. Due to the geometry and structural integrity of air ducts, flexibility and light weight are required key features. A hybrid wheeled tensegrity design would add speed and efficiency to the features of the robot. Basic work currently carried out in air ducts includes inspection and maintenance. However, in the near future, home automation, pioneered by companies like Apple, Google, and Amazon, will require the installation of cable terminals to power automated air vents. Being able to access areas through complex duct networks will continue to become more relevant.

## Literature review

Tensegrity structures have advantages and benefits. A list by Skelton et al. [5] includes: stability, efficiency, deployability, easy tunability, reliability to be modelled, facilitation of high precision control, promoting integration of structure and control, and being inspired by biology. Other features proposed by Komendera [6] include reconfigurability, failure tolerance, simplicity of design, and ease of modelling.

Tensegrity is an ever-expanding field. Studies on tensegrity structures have been “widely carried out since 1970s, mainly about form-finding, static stability, and dynamic and control” [7]. The work of finding new topologies and morphologies is called form-finding. This is a major subarea inside tensegrity robotics with several papers covering it. However, some researchers believe there are only a “few effective analytical methods for discovering tensegrity geometry” [8]. Williamson and Skelton [9] established a general class of tensegrity structures, defined the topological structure necessary to achieve tensegrity, and provided necessary and sufficient conditions for equilibrium. Motro [10] provided a description of “morphogenesis of tensegrity since earlier cells to present”. Then, he presented a numerical model to create more complex systems. Rieffel et al. [11] introduced an evolutionary algorithm which produces large tensegrity structures. They also demonstrated its efficacy and scalability relative to previous methods. They claim that those “techniques have produced the largest and most complex irregular tensegrities known in the field”. On the other hand, Hernàndez and Mirats-Tur [12] did an important job to complement the above-mentioned studies. They presented a method to detect and avoid “internal collisions between structure members and external collisions with the environment”. Once there is a defined topology of a tensegrity structure and an initial stable placement, Micheletti and Williams [13] offered a “simpler approach for discovering the range of feasible geometries”; also known as morphologies. Tensegrity is widely adopted by nature. Many researchers have taken inspiration from examples such as a vertebral column [14–17], a joint elbow [18, 19], ant colonies [7], caterpillars [8], and fish [21] in order to produce new topologies called biotensegrity structures. They imitate their counterparts in nature and grasp some of the properties they possess.

After the topology and morphology are defined, mathematical models can be created. A static analysis review was carried out by Hernàndez and Mirats [4]. However, the dynamics were first studied by Motro et al. [22]. A model of tensegrity robotics based on Euler–Lagrange equations of motion was described by Komendera [6]. Mirats-Tur et al. [23] developed dynamic equations for a 3-bar tensegrity mobile robot. Cefalo and Mirats-Tur [24] proposed a new dynamic model for a class-1 tensegrity system based on quaternions. The use of quaternions eliminates problems of singularities and allows to perform more precise calculations and simulation as they do not need to use trigonometric functions for the representation of angles. This is a lesser developed area, there are only very few morphologies analytically studied, being the most predominant the 3-strut prism as in the works of Skelton and Oliveira [25].

When efficiency is mentioned as one of the features of tensegrity robots, it refers to this potential energy stored as tension inside the structure itself. However, this efficiency does not relate to locomotive movement. All research papers studying locomotion by means of tensegrity do not measure efficiency in terms of distance covered over energy consumed. However, at least velocity is mentioned in the works of Friesen et al. [26] who stated that DuCTTv2 moves at 1.4 cm/s and Hustig et al. [18] whose simulation of a robot travels at 3.8 cm/s.

Although there is no mention of energy consumption. Paul et al. [27] introduced the idea of a legged robot based on tensegrity. Later, Böhm worked extensively and produced various papers (2012–2014) about locomotion by means of tensegrity mechanisms. His prototypes are capable of uniaxial rolling and movement in a plane with a combination of tip over and rolling. The approaches are not centred on locomotive efficiency, and therefore, there are no data related to velocity or energy consumption. Rovira and Tur [28] derived dynamic equations of motion based on kinetic energy and potential energy expressions included in Euler–Lagrange equations. They simulated path trajectory including ground and friction into their model. Shibata et al. [29] described the design of a tensegrity robot capable of crawling and proposed future designs that are able to jump. Bruce et al. [30] developed a compliant and distributed tensegrity robotic platform for planetary exploration: SUPERball. The morphology of the robot is an icosahedral. It is a practical application of tensegrity robotics that NASA sponsors. The paper does not focus on the locomotive part of the robot, only design. Sabelhaus et al. [31] show the construction aspect of SUPERball prototype. Friesen et al. [32] developed another practical application, a tensegrity robot capable of climbing vertical ducts. Friesen’s robot, called DuCTT (duct climbing tetrahedral tensegrity), comprises two tetrahedral frames connected by eight actuated cables. The robot holds to the duct by applying pressure against the wall with one linear actuator in each tetrahedron. It advances by moving one frame relative to the other. Researchers discuss in the paper the inverse kinematic control strategy used to actuate the robot. They point out that wheeled duct climbers can move quickly but “have difficulty overcoming sharp corners or other irregularities” [32] and that the use of rigid joint legs requires heavy actuators to achieve vertical ascent. Tests on this robot were included in an updated paper, but the robot is still not able to turn into a new branch. Friesen showed computer simulated work on NTRT (NASA Tensegrity Robotics Toolkit) developed by NASA to facilitate rapid design and control of tensegrity robots. Hustig et al. [18], Mirletz et al. [16], Lessard et al. [20], SunSpiral et al. [33], Bruce et al. [30], and Sabelhaus et al. [31] have also used NTRT. This software has been validated with prototypes.

## Criteria of evaluation

Designing a hybrid wheeled tensegrity robot, to the authors’ knowledge, has never been attempted before. Therefore, some criteria of evaluation need to be established beforehand in order to be able to interpret the results of this study.

### Antagonistic tensional elements

The first criteria developed were meant to classify tensional elements in two groups: elements that produce contraction of the structure and elements that produce Expansion.

**Fig. 3:**
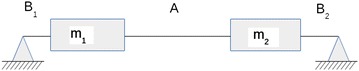
Antagonistic tensional elements. A brings m_1_ and m_2_ together, while B_1_ and B_2_ separates them

Consider the system shown in Fig. 3 comprised of two masses m_1_ and m_2_ joined by cable A and attached to fixed points by cables B_1_ and B_2_ at each side of the masses. The tension in cable A is Ta, and the tension in cables B_1_ and B_2_ is Tb.

**Fig. 4:**
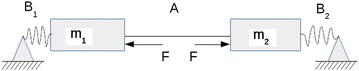
Tensional element of expansion. Cables B_1_ and B_2_ relax when F tries to expand the system

The first situation presented (Fig. 4) is the system affected by force F applied in the direction pointing towards the centre between the two masses. When this force is applied, tension in cable A is reduced by a magnitude F and tension in cables B_1_ and B_2_ is increased by a magnitude F. If the cables are elastic and force F big enough to stretch them, force F would bring masses m_1_ and m_2_ closer together. Force F is replacing cable A in bringing the masses together. Thus, it can be said that cable A brings masses m_1_ and m_2_ together. Tension in cable A is a tension of Contraction.

**Fig. 5:**
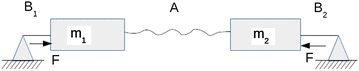
Tensional element of contraction. Cable A relaxes when F tries to contract the system

The second situation presented (Fig. 5) is the same initial system affected by a force F in the direction opposite to the centre between both masses. When this force is applied, tension in B_1_ and B_2_ is reduced by a magnitude F and tension in cable A is increased by a magnitude F. If the cables are elastic and force F big enough to stretch them, F would take masses m_1_ and m_2_ apart from each other. Force F is replacing cables B_1_ and B_2_ in separating masses m_1_ and m_2_. Tension in cables B_1_ and B_2_ is a tension of Expansion.

**Fig. 6:**
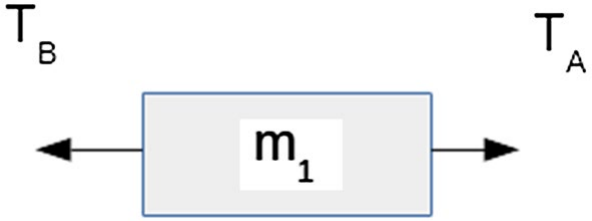
Antagonistic tension. Tension T_A_ and T_B_ are mutually antagonistic

In Fig. 6, mass m_1_ is in stable equilibrium in the horizontal direction due to a tension of contraction T_A_ and a tension of expansion T_B_. Creating a stable equilibrium through tension is the central concept of tensegrity.

### Strong and week tensional topology

**Fig. 7:**
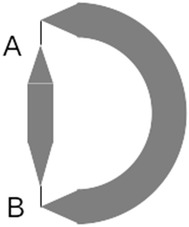
Weak tensional topology. Tension in cables A and B provides strong stability in vertical direction but poor stability in horizontal direction

**Fig. 8:**
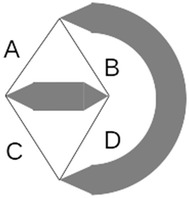
Strong tensional topology. Tension in cables A, B, C, and D provides strong stability in vertical and horizontal direction

Tensegrity systems are made rigid by tension only, and therefore, the direction that tension is applied is central to their design. The system topology shown in Fig. 7 has strong stability in the vertical direction but poor stability in the horizontal direction. This is due to the fact that cables A and B are aligned with the vertical direction and perpendicular to the horizontal direction. The system topology shown in Fig. 8 has strong stability in all directions in the plane. This is because cables A, B, C, and D have a projection in every direction of the plane.

When designing a tensegrity system or a transitional region, this criterion has to be taken into consideration in order to have a stable system in all directions. In general, each structural element must be held by tensions that, considered together, have balanced projections on to all basis axes of the coordinate system. This implies a design principle that we will see is repeated throughout tensegrity structures: each structural node of the system must have *at least three cables in tension* attached for true three-dimensional stability.

## Review of tensegrity systems

An extensive study of different topologies and morphologies was carried out as part of the research work needed to design a hybrid wheeled tensegrity robot. This was an essential step in order to select the tensegrity structures that best fulfilled the design requirements. All tensegrity systems shown in this chapter were built by the author in order to gain a deep understanding of the capabilities, advantages, and disadvantages of each of them relative to a duct travelling robot.

The area of tensegrity that studies the use of linear elements to form three-dimensional tensegrity structures is what the author calls Linear Tensegrity. This is where linear rigid elements, normally called struts or rods, and linear elastic elements, normally called strings or cables, are combined to form structures in two and three dimensions. Three-dimensional structures built with linear elements are the most common area of study in tensegrity. Planar tensegrity would include the use of two-dimensional elements; this was not covered in this exploration due to the short time available.

The first half of this chapter is introductory to the mechanics of tensegrity systems. It is focused on the study of the triangular prism. “Almost all tensegrities can be seen as variations on tensegrity prisms” (T. Flemons, personal communication, 2017). It starts with a line segment, and it builds up a structure in steps until a triangular tensegrity prism is achieved. It is important to understand these basic structures in order to be acquainted with the more complex ones presented later in the chapter.

The second half of this chapter continues dissecting tensegrity structures as it increments the complexity of them. It also shows how these structures relate to a potential design for a robot and discusses about advantages and disadvantages of each one.

### Line segment

**Fig. 9:**
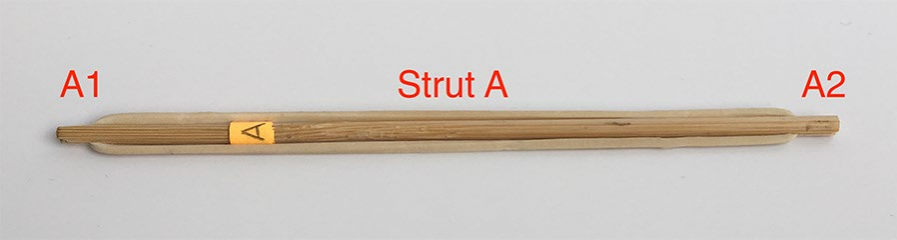
Tensegrity line segment. Comprises one strut and two cables

This is the basic building block for most regular structures. It is made with one strut and two cables on opposite sides. It should be noted that two cables are needed to have a properly balanced system with two cables under tension and one strut under compression. If it only had one cable, the system would generate momentum and shear forces. All tensegrity systems in this exploratory exercise will be built from this basic arrangement. Figure 9 shows a tensegrity line segment composed of one strut and two cables at each side.

### Square

**Fig. 10:**
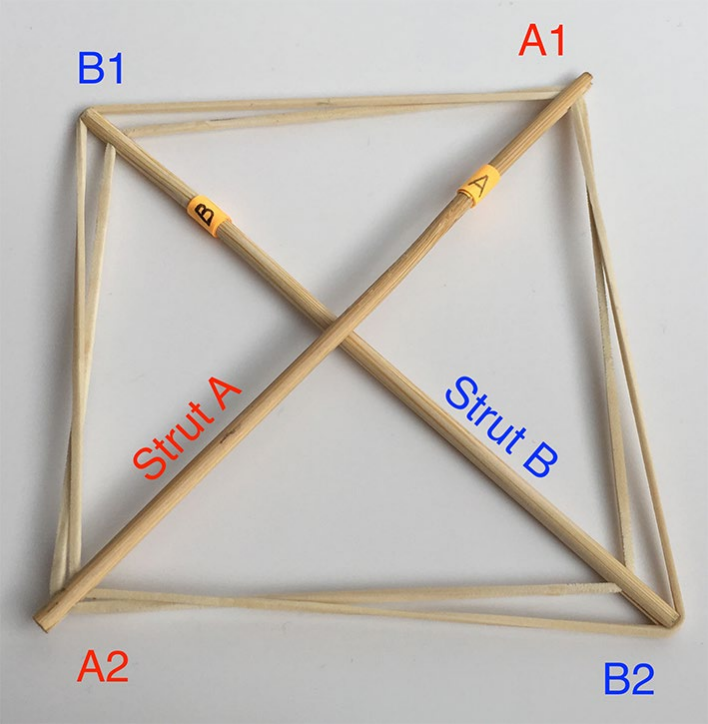
Tensegrity square. Comprises two struts and four cables

A square is made from two tensegrity line segments. It is constructed by inserting one tensegrity segment into another. Segment “B” is inserted in such a way that the ends of the strut are connected to the midpoint of the cables in segment “A”. Similarly, cables from segment “B” connect by the midpoint to the ends of the strut in segment “A”. The original cables in each segment are now considered split in two cables. Therefore, a square comprises two struts and four cables as shown in Fig. 10. It should be noted that all elements can be considered to be in the same plane for practical reasons even though the struts are intersecting in the middle. It should also be noted that as a planar structure, it has weak stability out of the plane if considered an element of a three-dimensional structure.

### Triangular prism

A triangular prism is constructed by inserting a third tensegrity segment “C” into the previously seen square. Also known as 3-strut prism, it is the simplest three-dimensional tensegrity structure and the very first one created and exhibited in 1921. It has three struts and nine cables. It also exhibits strong stability in three dimensions as the tension cables for each strut considered together have projections on all three-dimensional basis vectors, and three cables are attached to each node.

**Fig. 11:**
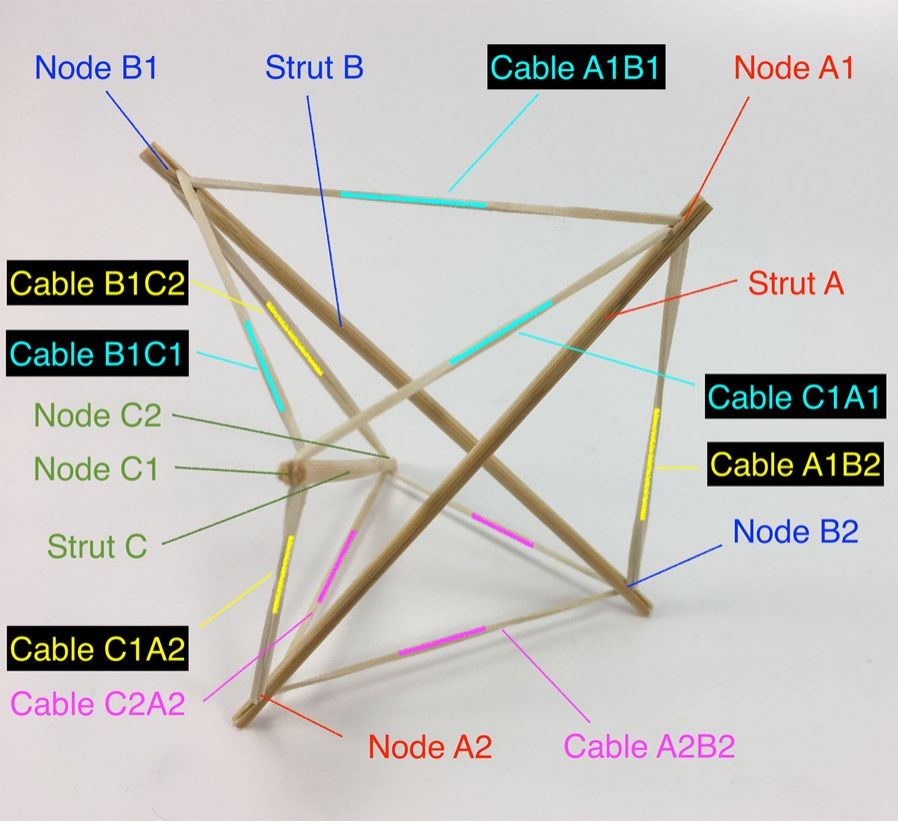
Tensegrity triangular (3-strut) prism. It is the simplest tensegrity three-dimensional topology. It comprises three struts and nine cables

**Fig. 12:**
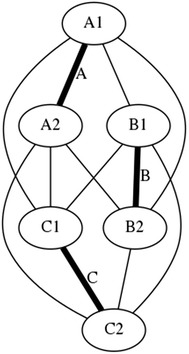
Graph of tensegrity triangular (3-strut) prism connections. This illustrates the symmetry of the structure with three cables connecting to each node

There is a particular nomenclature used in this analysis shown in Fig. 11. Each strut is marked with one colour: A is red, B is blue, and C is green. The end of each strut is called a node. Each node is named according the strut it belongs to. At the top of the prism, there are three nodes A_1_, B_1_, and C_1_. At the bottom of the prism, there are three more nodes A_2_, B_2_, and C_2_. Cables are named in relation to the nodes. At the top, there are three horizontal cables marked in cyan forming a triangle: A_1_B_1_, B_1_C_1_, and C_1_A_1_. Notice that the order is not important; A_1_B_1_ and B_1_A_1_ is the same cable. There are three vertical cables marked in yellow: A_1_B_2_, B_1_C_2_, and C_1_A_2_. At the bottom, there are three more horizontal cables marked in magenta forming another triangle: A_2_B_2_, B_2_C_2_, and C_2_A_2_. The connection of the six nodes is illustrated by the graph structure in Fig. 12, in which thick lines indicate struts A, B, and C and thin lines indicate cables in tension. This clearly indicates the symmetry of this structure, in which each node connects to three cables, and shows how such a structure can be generated mathematically.

As part of this study, three intermediate structures between the tensegrity square and the triangular prism were devised to help investigate the properties of a three-dimensional tensegrity structure and show what results if the symmetry mentioned above is broken. In other words, these are artificial transitioning state topologies between a 2D and a 3D tensegrity structure that help understand how a 3D tensegrity structure is able to stand; its internal mechanics and the relationship between its elements. The mechanics of such structures are integral to the performance of a tensegrity-based robot and also illustrate how a robot can be manufactured and transformed between different topologies.

**Fig. 13:**
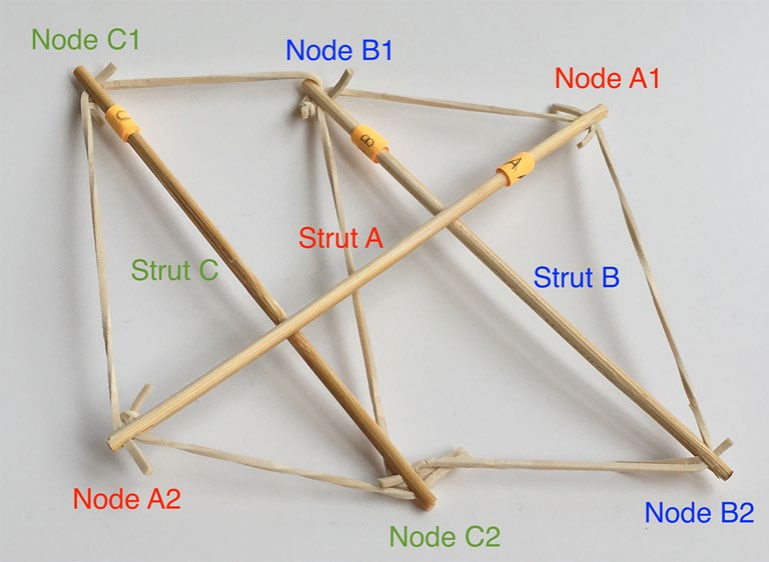
Single plane. Strut A allows the separation of struts B and C. If strut A is removed B and C would join

**Fig. 14:**
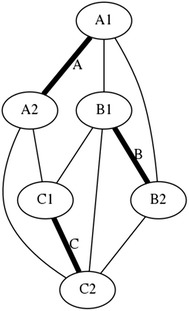
Graph of connections for Single plane. This illustrates the asymmetry of the new structure
- *Dual plane with three intersecting struts* This intermediate state topology, shown in Fig. 15, is achieved by eliminating cables C1A1 and A2B2, and adding cable C1B2. The structure collapses into two intersecting planes. The first plane is formed by struts A and B, the second plane is formed by struts A and C. Strut A is the inserted strut separating struts B and C. The same case can be made to explain that all cables are elements of expansion and contraction simultaneously. Although in this topology one top horizontal cable, one bottom horizontal cable, and two vertical cables are elements of expansion. This structure remains three-dimensional although all three struts are in direct contact. This structure illustrates how changing to different topologies can transform the structure of a robot, in this case to a plane-oriented structure that could be used to obtain specific body orientations (Fig. 16).

- *Single plane* This intermediate state topology, shown in Fig. 13 and achieved by eliminating cables C1A1 and A2B2, has become a two-dimensional structure. It can be seen that cables A1B1, A1B2, C1A2, and C2A2 are tension elements of expansion. They separate struts B and C. In the same way, cables B1C1, B1C2, and B2C2 are tensional elements of contraction. Expansion elements transmit the tension to the rigid element strut A which is the inserted strut that allows separation of struts B and C. If strut A is removed struts B and C would join and overlap as effectively a one-dimensional structure. In this topology, two top horizontal cables, two bottom horizontal cables, and two vertical cables are elements of expansion. Similarly, one top horizontal cable, one bottom horizontal cable, and one vertical cable are elements of contraction. If the topology is changed so that now strut B is the inserted strut, it would allow the separation of struts A and C, the same number of cables will be elements of expansion and contraction. Therefore, it is demonstrated that each cable in the prism topology is an element of expansion and contraction simultaneously. This also demonstrates how such a prism could be assembled or “unwrapped” in a robot’s structure. A graph illustrating the broken symmetry of the structure is shown in Fig. 14.

**Fig. 15:**
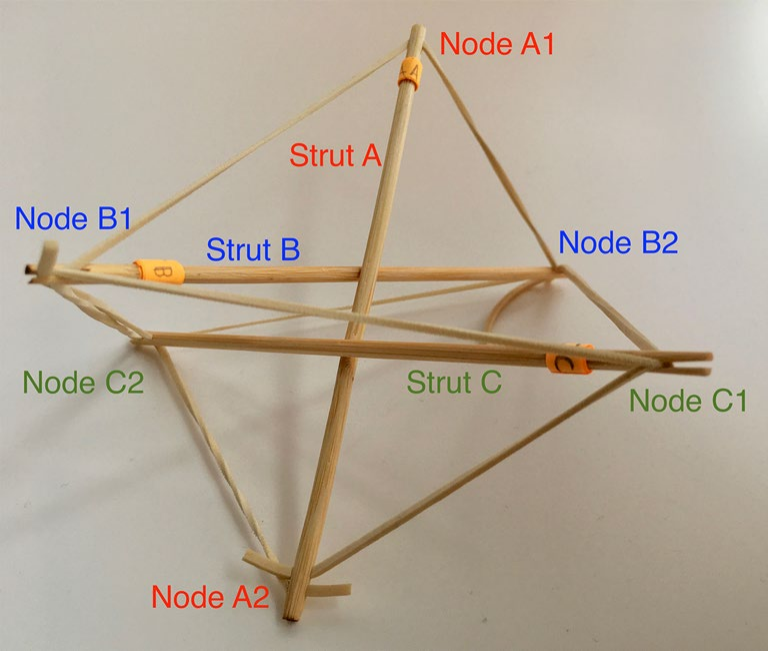
Dual plane with three intersecting struts. Strut A allows the separation of struts B and C. If strut A is removed B and C would join

**Fig. 16:**
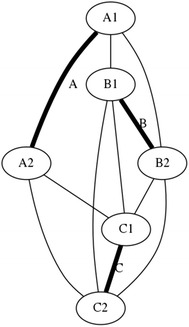
Graph of connections for Dual plane with three intersecting struts
- *Dual plane with two intersecting struts* This intermediate state topology, shown in Fig. 17, is achieved by eliminating cables B1C1 and A2B2. The prism is broken into two planes. The first plane is formed by struts A and C, and the second plane is formed exclusively by strut B. This state is unstable as strut B can rotate along nodes A1 and C2. The eliminated cables are elements of contraction. The remaining cables attached to strut B are elements of expansion. Thus, it can be appreciated how they interact to keep strut B stable. This state is essentially formed by a square tensegrity (struts A and C) and a segment tensegrity (strut B) added to one side of the square, and illustrates how stability can be controlled by varying the elements of a tensegrity robot’s structure (Fig. 18).

**Fig. 17:**
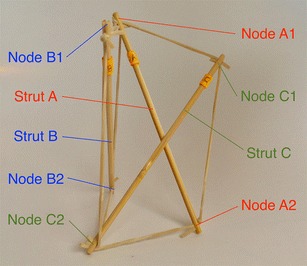
Dual plane with two intersecting struts. Elements of contraction to strut B were eliminated. Strut B is unstable

**Fig. 18:**
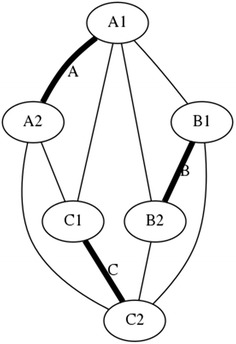
Graph of connections for Dual plane with two intersecting struts

### Square prism

**Fig. 19:**
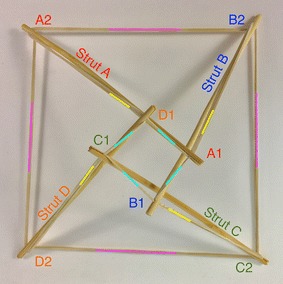
Square prism. Comprises four struts and twelve cables. Top view

**Fig. 20:**
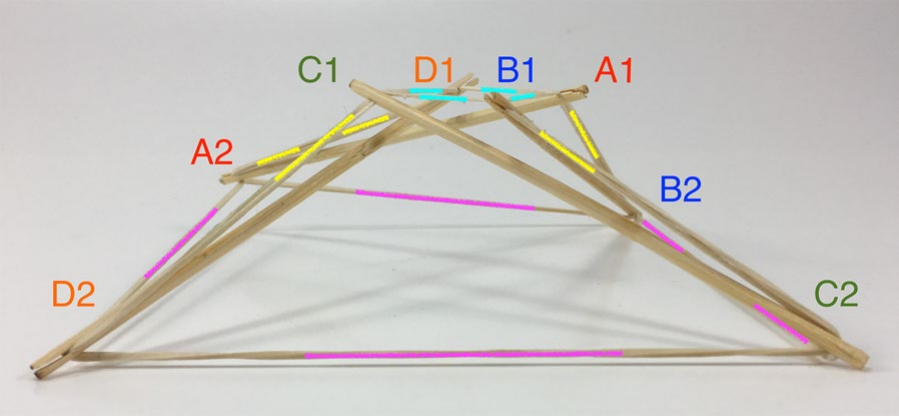
Square prism. Side view

A square prism is comprised of four struts and twelve cables. A square prism and any other prism follows the same relationship found for the triangular prism. Top horizontal cables are marked in cyan, vertical cables are marked in yellow, and bottom horizontal cables are marked in magenta as shown in Figs. 19 and 20. All prisms are made by adding one or more struts to the triangular prism.

**Fig. 21:**
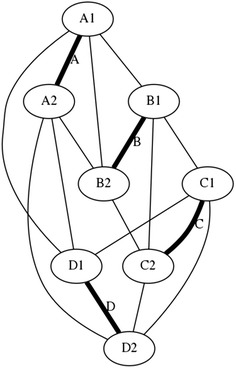
Graph of square prism connections

In the context of a duct travelling robot, the square prism is better fit for a square duct cross section where each strut could be pressed against each corner of the square section in the duct. The morphology shown has the bottom square with larger dimensions than the top square. This could be beneficial for expanding a narrow axle into a much bigger structure, as a joint between a square bar and another tensegrity structure or to reach out to the walls of a bigger ducting. A graph of the structural connections is given in Fig. 21.

### Octagonal prism

**Fig. 22:**
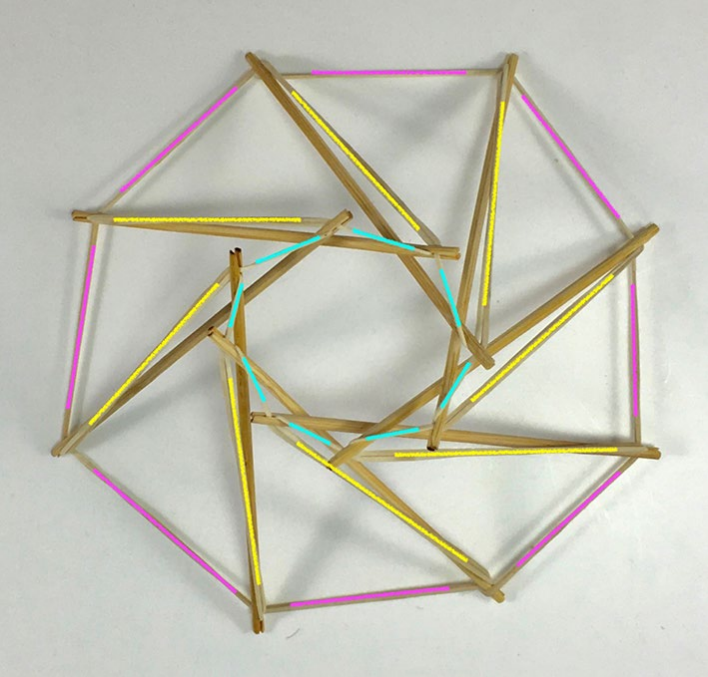
Octagonal prism. Comprises eight struts and twenty-four cables. Top view

**Fig. 23:**
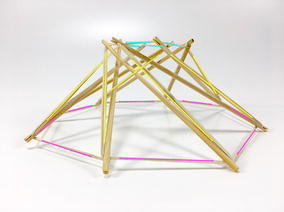
Octagonal prism. Side view

**Fig. 24:**
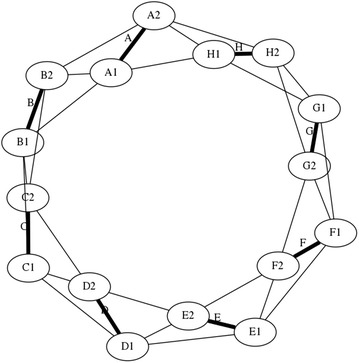
Graph of octagonal prism connections

Comprised of eight struts and twenty-four cables, the octagonal prism, shown in Figs. 22 and 23, is also well suited for a square ducting. Two struts could press into each wall of the duct. By expanding and contracting one of the octagons, the robot could adapt to different duct sizes. The end of the struts could be attached to wheels in order to support the structure inside the duct. This topology could also be used as a vacuuming tool. If the struts are made of piping and hooked to hoses converging into one hose, this tool could vacuum the walls of different sizes of square duct. A graph of the connections in the octagonal prism is given in Fig. 24, which also provides labels of all elements.

### Icosahedron

**Fig. 25:**
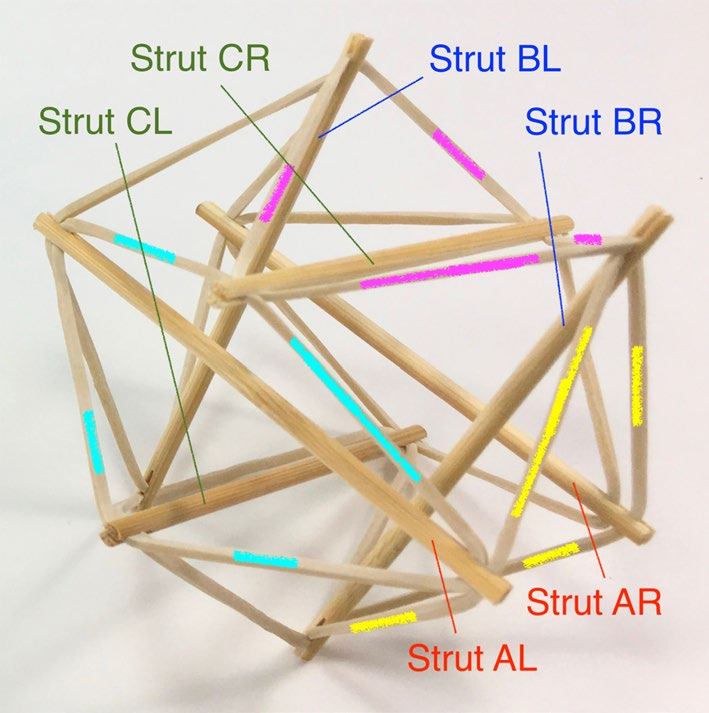
Icosahedron. Comprises six struts and twenty-four cables

The expanded octahedron or icosahedron has six struts and twenty-four cables. All 6 struts can be organised in three groups: A, B, and C. Each group has of two parallel struts R and L (right and left). Therefore, all struts can be labelled AR, AL, BR, BL, CR, and CL as shown in Fig. 25. Cables can also be organised in six groups. Each group has four cables that resemble the shape of a rhombus. Three groups of cables were marked in cyan, magenta, and yellow. Adjacent to the three rhombi marked with colours, there is a triangle formed by one cable of each colour (see longer marks). There are eight of these triangles in the icosahedron.

**Fig. 26:**
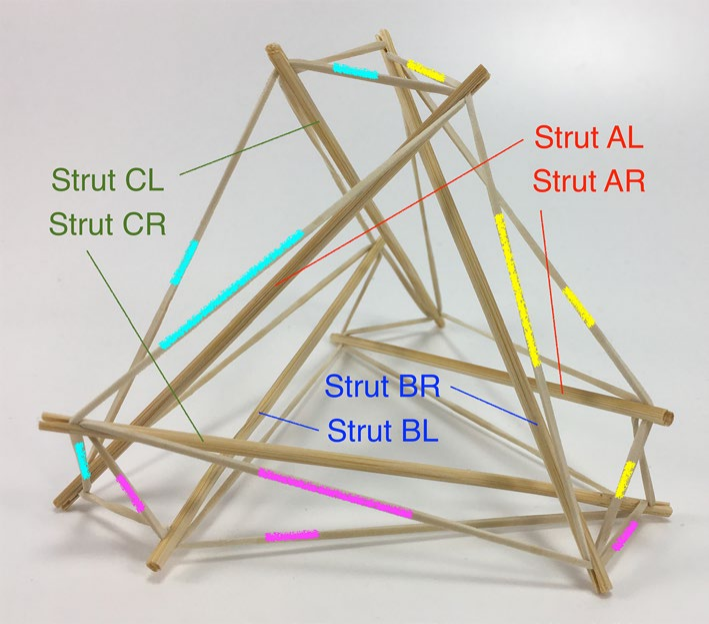
Truncated tetrahedral. Comprises six struts and twenty-four cables

**Fig. 27:**
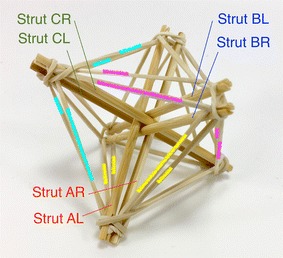
Octahedron. Comprises six struts and twenty-four cables

**Fig. 28:**
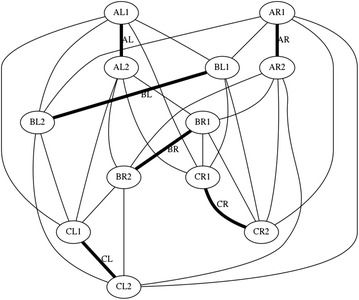
Graph of icosahedron connections

The morphology of this topology can be changed into a truncated tetrahedral (Fig. 26) by simply changing the length of the cables. If the parallel struts of the icosahedron are brought together it forms an octahedron (Fig. 27) and therefore the name of expanded octahedron for the icosahedron. The same marking and labelling has been kept in all three Figs. 25, 26, and 27 to emphasise that this is the same topology although it looks very different in each of the morphologies shown. A graph of the connections between the elements of the icosahedron is given in Fig. 28.

The icosahedron resembles a ball when all struts and cables are the same length. NASA is developing a robot with this morphology that is capable of rolling as method of locomotion [31]. In relation to a duct travelling robot, this could be useful as a joint or coupling. As it will be explained later a robot could be made of several localised tensegrity systems, the icosahedron is suited for this application.

### Octahedron

Formed by joining the parallel struts of the icosahedron together, Fig. 27. This morphology could also be used as an intermediate state between a tensegrity and a solid. Although this is not as flexible as other tensegrities as it has all struts joined in the centre. If two parallel struts are extended out of the localised tensegrity area, they could connect with another localised tensegrity; the same way the Ulna and Radius connect the elbow with the wrist. This would provide structural support and mobility. The connection graph of the octahedron is the same as that of the icosahedron, but with the addition of cables AL1AR1,AL2AR2, BL1BR1, BL2BR2, CL1CR1, and CL2CR2.

### Truncated morphologies

This is a class on its own right. The number of struts required is the same as the number of edges, and the number of cables required is equal to three times the number of vertices plus the number of edges.

**Fig. 29:**
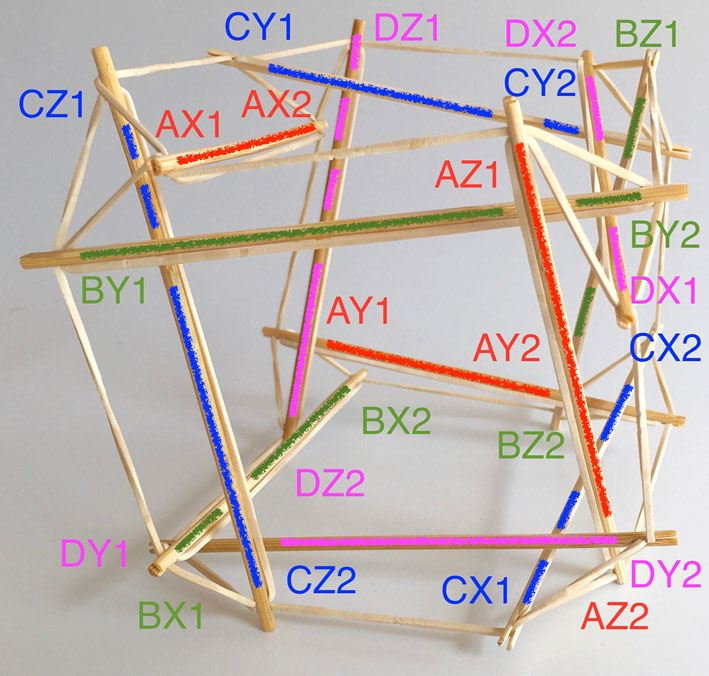
Truncated cube. Comprises twelve struts and thirty-three cables

The truncated tetrahedral, Fig. 29, can be made by varying the length of the cables in the icosahedron. Both are two morphologies of the same topology and share the same connection graph. Although this tetrahedral was built with segments made of one strut and two cables (a total of 24 cables), it can also be constructed with segments made of one strut and one cable (a total of 18 cables).

This structure could be used as flexible chassis for a three-wheeled robot. In a duct travelling robot, this structure could be used as a nose at the front of the robot to carry an instrument. The nose can be pointed in different directions by changing the tension in the cables. It can also be used as a tail to stabilise the robot when it needs to change direction into a new branch.

**Fig. 30:**
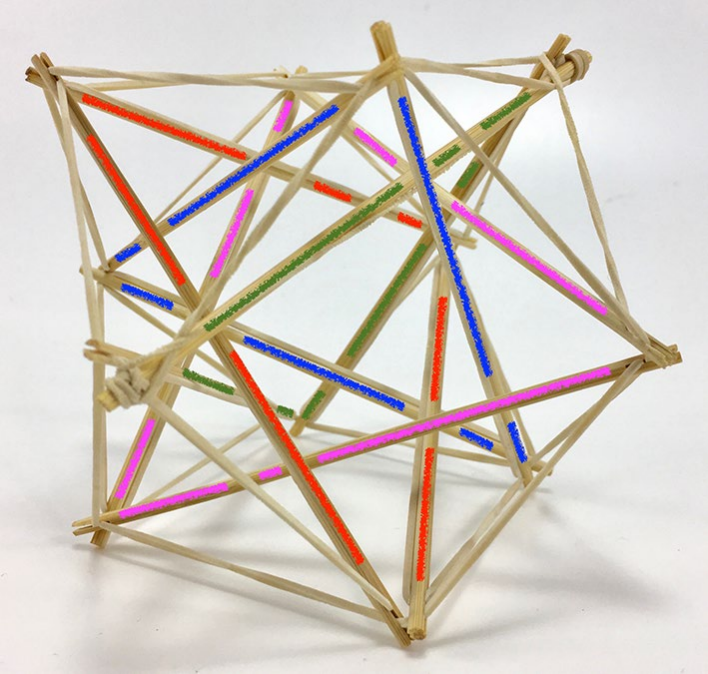
Four intercepting triangles morphology. Comprises twelve struts and thirty-three cables

A truncated cube, Fig. 29, has 12 struts and 36 cables. As an alternate modelling method, we can consider this structure as having struts oriented in the X, Y, and Z directions and group these struts into three groups of X, Y, and Z elements as distinguished by colour in Fig. 30. This structure can be used as suspension for wheels in a robot. The chassis can be raised and lowered by changing the tension in the cables. The truncated cube can also be used as a coupling in a soft transitional region (explained further down in this paper).

**Fig. 31:**
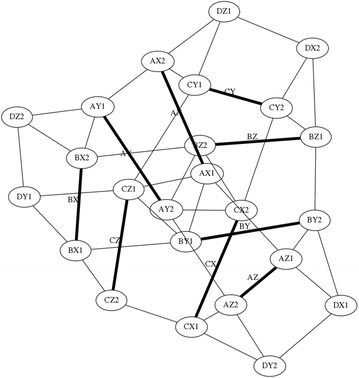
Graph of truncated cube connections

When the ends of the struts on each side of the cube are joined closer together a different morphology comprised of four intersecting triangles is made as shown in Fig. 30. Both Figs. 29 and 30 were colour marked to appreciate how the same struts have been displaced, and Fig. 31 shows a graph of connections.

**Fig. 32:**
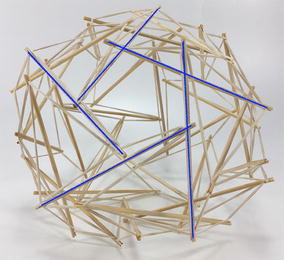
Truncated dodecahedral. Comprises thirty struts and ninety cables

Following the same pattern, a truncated dodecahedral, Fig. 32, can be built with 30 struts, 90 cables and having five-sided faces. This structure behaves essentially as a ball and is very complex to model and not of practical use for the purposes of the duct climbing robot in this paper.

### Spiral vertebral mast

**Fig. 33:**
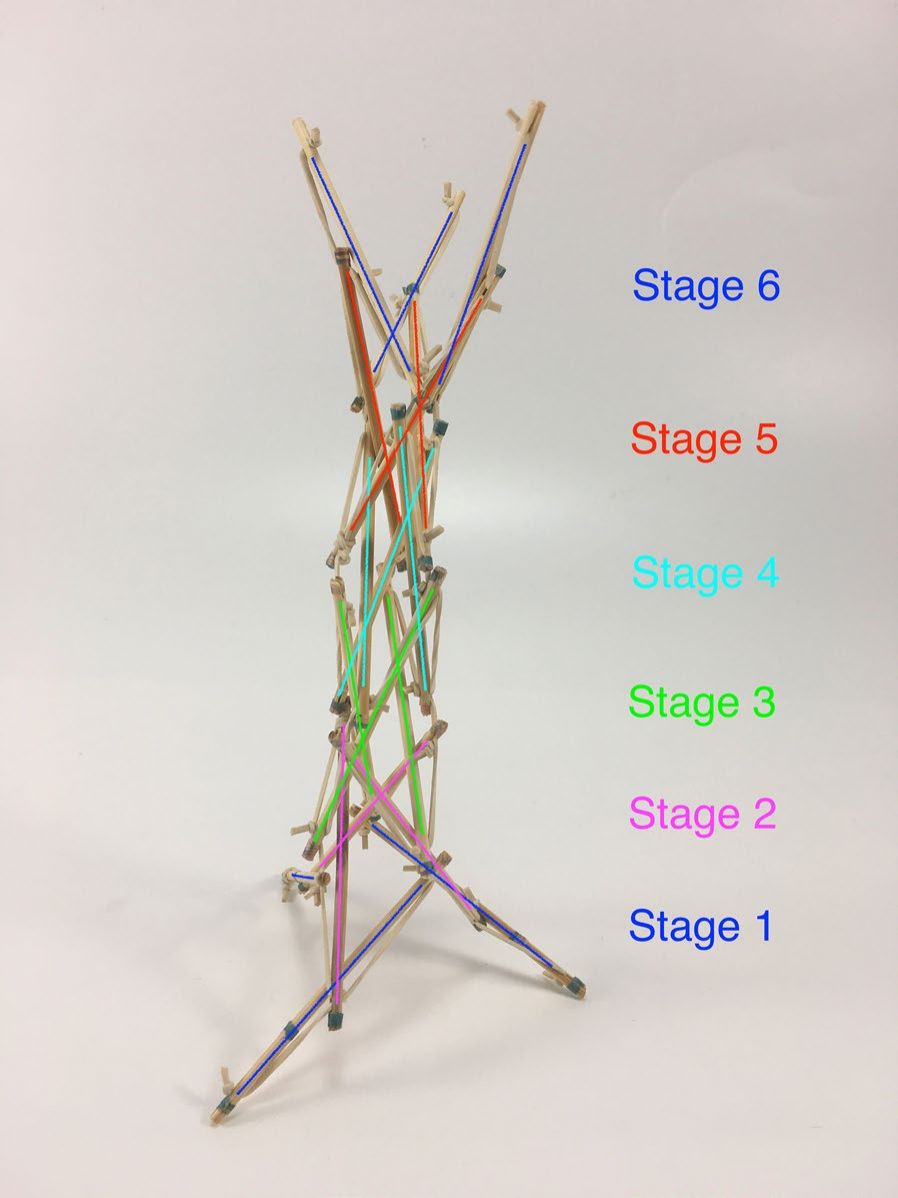
Spiral tensegrity mast. Minimal topology

**Fig. 34:**
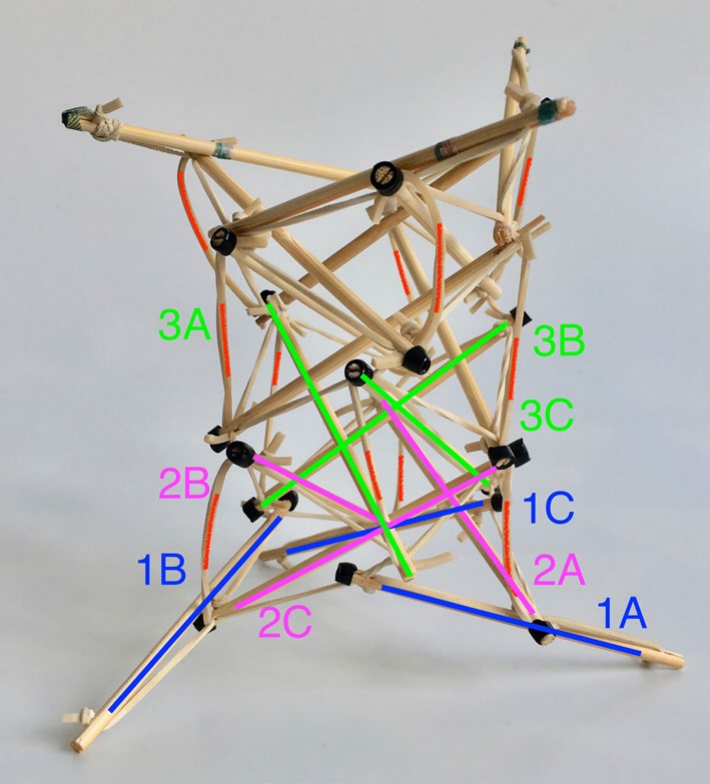
Spiral tensegrity mast. Antagonistic tensional topology

**Fig. 35:**
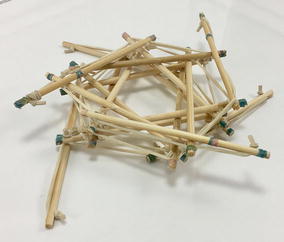
Spiral tensegrity mast. Collapsed state. Achieved by placing a glass on top of the mast

**Fig. 36:**
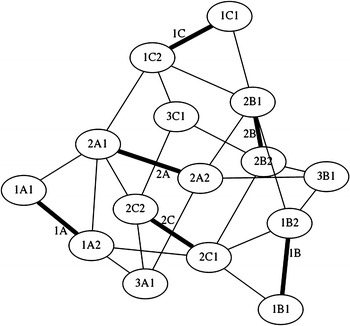
Graph of minimal three stage topology of spiral tensegrity mast

While the structures considered up until now are illustrative of tensegrity principles and show how such structures could be of use in robotics, we now consider structures that are less based on geometric primitives and more on repeated structures found in nature. The first structure we consider in this area, the spiral vertebral mast, is built in stages. In this model, each stage has three struts that attach to the cable midpoint of the previous stage. The orientation of each stage changes, if the first stage is oriented clockwise, the second stage is oriented counter clockwise. This structure, designed by Tom Flemons, can be built to be as long as desired following the same pattern. At its minimal topology, it only has tensile elements of contraction as shown in Fig. 33. This is why in a state of equilibrium, with no other external forces, it looks shrunk in its radial direction and it does not take a great volume in space. The tension forces are almost aligned axially which creates a weak tensional morphology in the radial direction. This could be corrected by adding antagonistic tensional elements as highlighted in Fig. 34. To make use of the staged nature of the mast structure, we enumerate the elements as A, B, and C at each stage, with prefixes to indicate the stage number starting from the bottom. Hence, the first stage is composed of struts 1A, 1B, and 1C, while the second stage is ordered in the opposite direction as 2A, 2B, and 2C to reflect the dual-spiral structure. The spiral vertebral mast can be collapsed along its axial direction as shown in Fig. 35 and will recover its original form when the external force applied is eliminated. The structure can be built with three or more struts in each state following the same pattern. Figure 36 shows a graph of the connections for two complete stages of the structure connected to a third, with additional stages being added following the same pattern. Note that nodes 1A1, 1B1, and 1C1 are supporting the structure on the ground and nodes 3A1, 3B1, and 3C1 represent connection points to the next stage of the structure, hence the low number of cable connections.

### Tetrahedral vertebral mast

**Fig. 37:**
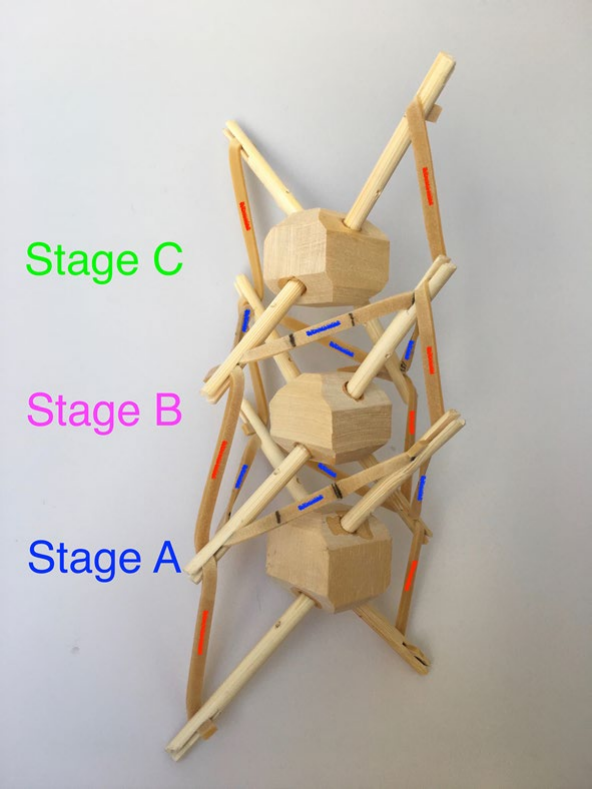
Tetrahedral vertebral mast. Three stages fully assembled

**Fig. 38:**
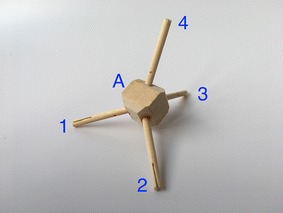
Tetrahedral vertebral mast. One isolated stage

**Fig. 39:**
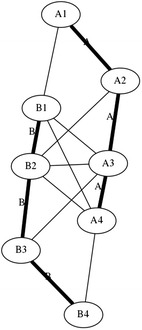
Graph of two stages connected in tetrahedral vertebral mast

The mast presented in Fig. 37 comprises three rigid elements joined together by eight cables. This structure is one of the few examples in which the element of contraction and expansion are well defined. Horizontal cables (marked in blue) are exclusively tensional elements of expansion, while vertical cables (marked in red) are exclusively tensional elements of contraction. As each stage of this mast is composed of only one rigid element, the elements are denoted by letters alone. Each rigid element comprises four struts rigidly joined together in the centre with nodes numbered as shown in Fig. 38. This structure, also designed by artist Tom Flemons, can be built with as many stages as needed. It is possible that the rigid tetrahedral can generate shear forces at the centre of the rigid elements. This is something that could be further evaluated. With the materials used in these models and the prototypes, the rigid element did not fail at its core. It was more likely to break at the rubber band connection wedge. A graph of two stages connected in a tetrahedral vertebral mast is shown in Fig. 39 which shows the relatively simple connections between two elements that can be easily repeated in a large structure, though it is important to remember that the geometry of the rigid elements that is not illustrated in a simple graph is essential to providing the tensional stability in this kind of tensegrity structure.

**Fig. 40:**
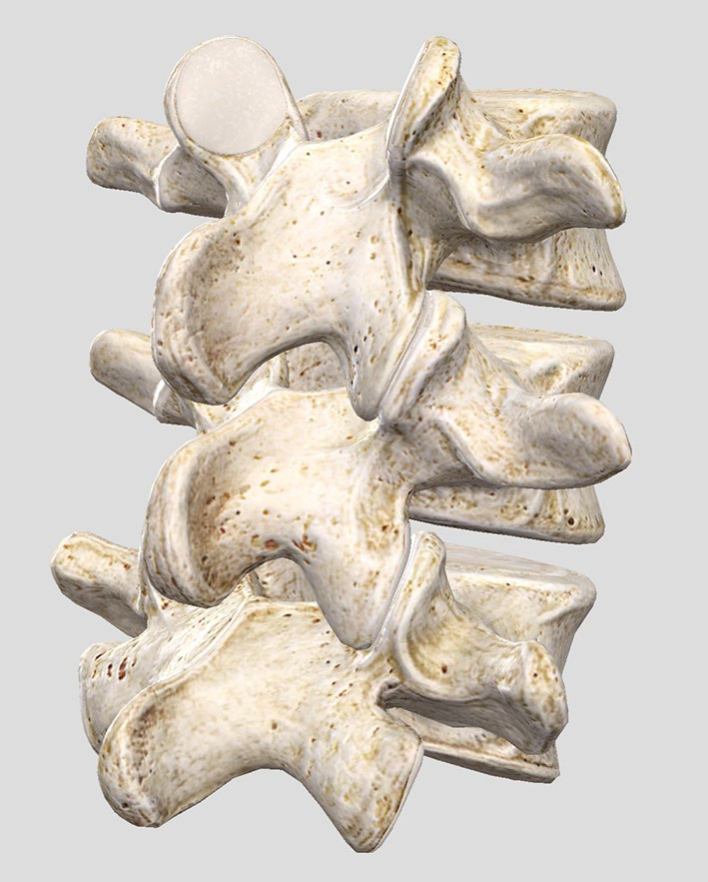
Tetrahedral vertebral mast. Animation of part of vertebral column [34]

This structure, in a way, is a simplification of the vertebral column shown in Fig. 40; each rigid element is one vertebra. In this analogy, there is no vertebral body and it is replaced by a strut as if the vertebra had two spinous processes. The two transverse processes are replaced by two struts as well.

There is no representation for any of the four articular processes. They are omitted in this model which is one of the biggest differences. The forces produced by the vertebral body and articular processes are replaced by horizontal cables (marked in blue, Fig. 37). The connections between transverse processes and between spinous processes in each vertebra are identically substituted by vertical cables (marked in red, Fig. 37).

In summary, both the latter topologies, the spiral vertebral mast and tetrahedral vertebral mast, present similar characteristics that are greatly beneficial for a duct travelling robot:

- Vectorial shape. It can point to a determinate direction, and it has a clear axial direction.
- Built in stages. They can be added or subtracted to increase or reduce the robot’s length.
- Radial degrees of freedom. It allows the structure to yaw and pitch in an unlimited number of directions. It is virtually a universal joint.
- Axial degree of freedom. It allows the structure to roll.
- Axial displacement. The structure can extend and contract axially.
- Pattern repeated in the axial direction. Easy to manufacture and repair.

## Transitional regions

There are studies, like Motro [35], that address the problem of joining together various tensegrity structures to form more complex and bigger ones. However, to the authors’ knowledge, there are no studies that address the problem of joining together a tensegrity structure with a fixed one. Therefore, in order to connect a tensegrity structure with a traditional mobile fixed structure, a new methodology will need to be developed. The following is a series of concepts that need to be formalised in the area of tensegrity and robotics.

### Localised tensegrity

Typical tensegrity structures have cables attached to struts at their endpoints. It should be noticed that this is common to see but not a requirement that defines a tensegrity system. This arrangement is so spread among the research community that limits the creativity in the development of new topologies.

When observing the musculoskeletal systems of animals, it can be noticed that groups of muscles and tendons are localised into regions (i.e., shoulders, hips). Bones are the only elements to transcend those limits. It can be observed that the humerus extends beyond the shoulder and, similarly, the muscles in the shoulder area a located on one end of the humerus and do not extend up to the other end of it. In this case it can be considered that the rigid body acts as the transitional region between two localised tensegrity regions.

**Fig. 41:**
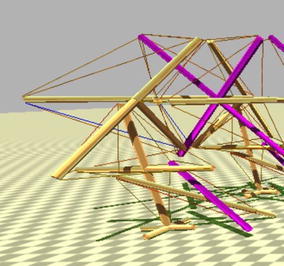
MountainGoat [18]. One tensegrity system

**Fig. 42:**
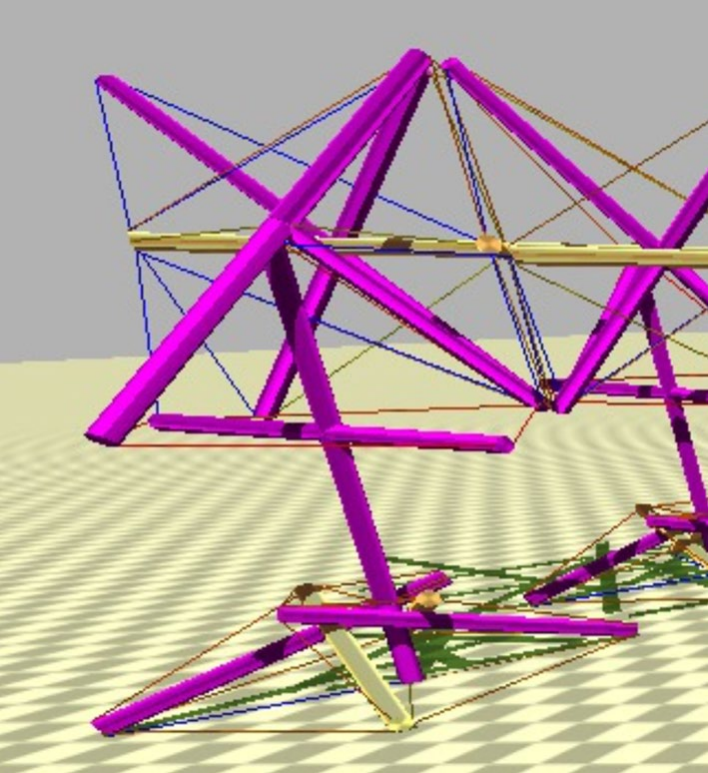
MountainGoat [18]. Two localised tensegrity regions

There are few exceptions to this paradigm that are starting to appear. Hustig et al. [18] did apply the concept of localised tensegrity in one of the iterations of his MountainGoat design without expressly acknowledging it in the paper. The following two figures show two versions of his design, one tensegrity system in Fig. 41 and two localised tensegrity regions in Fig. 42 (main body on top and foot at the bottom). As seen in the previous example, this concept can open the door to new more complex tensegrity systems comprised of multiple localised tensegrity regions. Each of them can be designed to perform specific functions within the entire structure.

### Hard transitional region

It is a transition made exclusively of rigid elements. This type of transition is linked to the concept of fulfilled pattern described below.

### Soft transitional region

It is a transition made exclusively of elastic elements. In order to be stable, it needs to have an antagonistic tensional setting: elements of contraction and elements of expansion. This type of transition is linked to the concept of unfulfilled pattern described below.

### Fulfilled pattern

The lumbar vertebrae are connected among themselves by three surfaces. One located at the vertebral body and two at the articular process. In total, a lumbar vertebra has six joints: three at the top and three at the bottom. This means that the fifth lumbar vertebra, the last one at the bottom, needs three surfaces of connection as well when joining the sacrum. The sacrum at birth is composed of five separate sacral vertebrae. Postnatally they fuse to form a single bone that is flattened anteroposteriorly and has a triangular shape when viewed from the front [36].

**Fig. 43:**
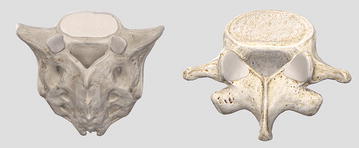
Sacrum and fifth lumbar vertebra. Top of sacrum is reproducing the pattern of top of vertebra [36]

This is where the principle of fulfilled pattern takes place. When connecting a chain of rigid elements of the same morphology to another element of different morphology, the morphology of the first needs to be satisfied by the second. That is, the sacrum needs to have the same top three connecting surfaces to receive the bottom three connecting surfaces of the fifth lumbar vertebra. Figure 43 shows how the sacrum follows the same pattern.

### Unfulfilled pattern

This concept can be observed in the connection of the scapula to the axial skeleton in the musculoskeletal system of the human body. The scapula attaches to the axial skeleton only with soft tissue. The scapula (shoulder blade) is a flat, triangular bone located along the posterior aspect of the thoracic cage. Seventeen muscles attach to the scapula; therefore, this bone is usually resistant to fractures [34]. Since the attachment of the scapula to the axial skeleton is exclusively made by muscles there is no requirement to imitate the form of the adjacent structure as in the previous case. Those seventeen muscles are arranged in a way that allows the body to (1) set an equilibrium state in which the scapula is at a particular location and (2) move the scapula around in a controlled way. This is a more complex joint to design. It certainly needs to be designed with antagonistic tensional elements and strong tensional topology.

## Robot design and prototyping

When using tensegrity structures to design mobile robots, designers face the following challenges [4]:

- *Static analysis of tensegrity structures* Find a stable configuration from a given topology or even design new topologies to achieve some desired results.
  - Form-finding methods
  - Static behaviour of tensegrity.
  - Rigidity and stability.
- *Dynamics and control of tensegrity* Plan trajectories and motions taking into account the advantages that tensegrity structures offer.
  - Traditional path planning algorithms.
  - Dynamic characterisation of tensegrity structures.
  - Node trajectory planning.

This paper is meant to cover the first three items from the list. As there is no previous academic work in robotics that uses coupling between solid bodies and tensegrity structures, it was necessary to first perform the introductory study shown previously.

The design of this prototype focuses on achieving two main requirements: (1) unprecedented flexibility for an air duct travelling robot and (2) unprecedented locomotive performance for a tensegrity robot. This section illustrates the development of the prototype and compares the features of this robot with the current state of the art.

### Work environment

Regarding geometric characteristics of air ducts, most common rectangular air ducts range from 100 mm × 200 mm to 150 mm × 300 mm and circular ducts, from Ø100 to Ø200 mm in households. The layout of ducts is mainly in a horizontal plane with turns right and left. However, there are also branches that go upward and downward. There are two requirements here: (1) a robot must adapt to the range of ducting sizes and (2) the robot must be flexible enough to turn not only left and right but also up and down or any other intermediate direction.

While requirement number one has already been solved by several designs, requirement number two has not. There are no robots in the market, to the authors’ knowledge, that can turn in any direction and are able to vertically climb. Therefore, requirement number two was the main focus of the design in this paper. The development process begun with the selection of the main tensegrity structure of the robot, followed by the design of the coupling from tensegrity to a solid body, and, finally, a model for the robot.

### Structure

Taking advantage of the flexibility of tensegrity structures to allow the robot to turn in every direction was the main requirement. All tensegrity structures are flexible, but not all of them have special flexibility in particular directions. Most of the structures tend to be omnidirectional and resemble the shape of a sphere.

However, there are two topologies that fit the needs of a duct traveller robot: The spiral vertebral mast and tetrahedral vertebral mast. They were chosen for reasons explained in “Transitional regions”. There was a slight modification to the original spiral mast: the structure constructed for the prototype was built with four struts on each stage for two reasons: (1) the robot will mainly turn up, down, right, and left, and (2) the robot fixed rigid body is a square bar. Therefore, four struts accommodate the geometric requirements better.

### Transitional region

**Fig. 44:**
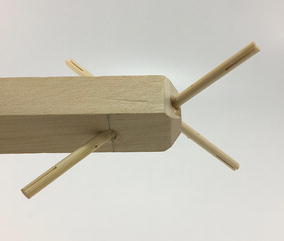
Hard transitional region. Tetrahedral vertebral mast

**Fig. 45:**
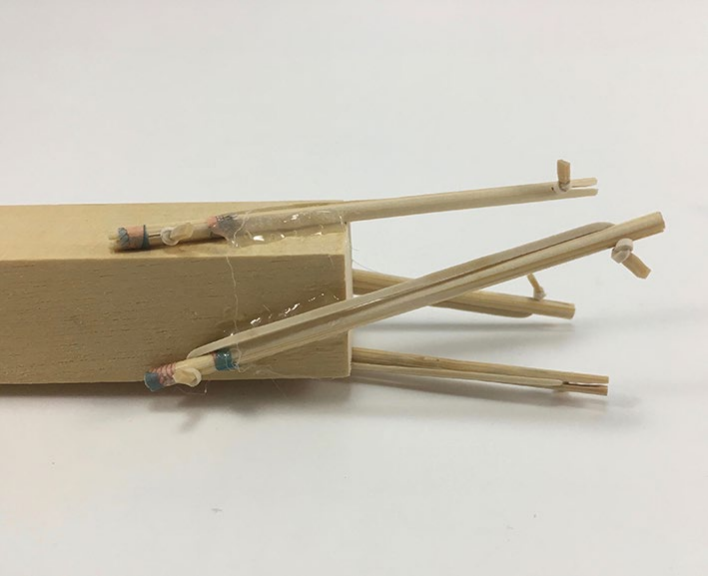
Hard transitional region. Spiral tensegrity mast

Initially, one prototype was made for each structure following the criteria of a hard transitional region and fulfilled pattern. For the tetrahedral vertebral mast (also called tensegrity spine), it was necessary to build all four struts and not only two as it was originally thought as shown in Fig. 44. For the spiral tensegrity mast (also called tensegrity mast), the construction process was carried out by placing the entire tensegrity mast next to the square bar, applying an external force to the four struts at the end of the mast until they touch the bar and gluing them while keeping the position they naturally acquired. When the glue dried the four struts did not belong to the tensegrity structure anymore but to the square bar as shown in Fig. 45.

**Fig. 46:**
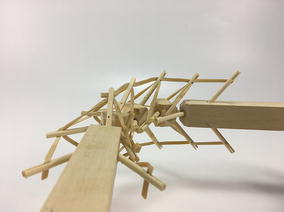
Hard transitional region prototype. Tetrahedral vertebral mast

**Fig. 47:**
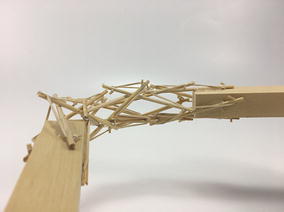
Hard transitional region prototype. Spiral tensegrity mast

**Fig. 48:**
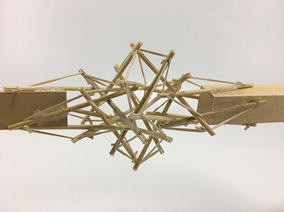
Spiral tensegrity mast. Collapsibility test

As can be seen in Figs. 46 and 47, both transitional regions imitate the pattern of the tensegrity structure they connect to (fulfilled pattern). Both tensegrities are capable of turning more than 90° in any radial direction, as well as rotating and translating in the axial direction. This concept worked well with the tensegrity spine shown in Fig. 46; it did not change any of the properties of the original tensegrity structure: axial translation, radial translation, axial rotation, and radial rotation remained the same. This design was acceptable, and no further development was needed for the structure. However, this concept did not work as well with the tensegrity mast shown in Fig. 47. It added rigidity to the original tensegrity structure and changed its mechanical properties. So much so that the structure was not able to fully collapse in the axial direction anymore. Comparing Figs. 35 and 48, both show a collapsibility test on this structure. In Fig. 35 the structure is free to fully collapse, while in Fig. 48 the structure is restricted and not able to fully collapse. However, collapsibility is not a requirement for this design, so the prototype was acceptable as well.

Two more prototypes of the spiral mast transitional region were made following the concept of soft transitional regions and unfulfilled patterns. However, the design was not tensional antagonistic and it had a weak tensional morphology. Therefore, the structure was unstable. More development would be needed to achieve both requirements in future prototyping iterations of this principle.

### Final model

**Fig. 49:**
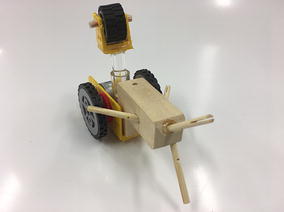
Motorised wheeled module. Front car

**Fig. 50:**
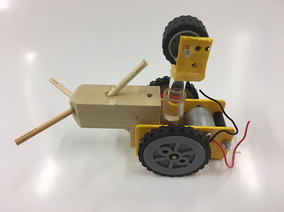
Motorised wheeled module. Rear car

**Fig. 51:**
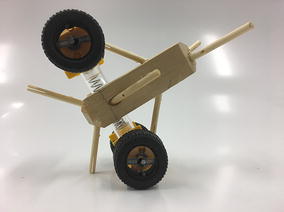
Non-motorised wheeled module. Middle section of the robot

The final prototype is the first wheeled tensegrity robot (WTR) ever built to the authors’ knowledge. It was made with readily available and recycled parts in the design laboratory at the University of Strathclyde. The final version comprises two motorised modules and one non-motorised module which are shown in Figs. 49, 50, and 51. Motorised modules have two wheels of Ø44 mm at the bottom and one wheel of Ø23 mm at the top. The top wheel presses against the wall of the ducting to generate traction. The motor runs on 4.5 V 0.41A at 4900 rpm. Overall dimensions are 110 mm × 58 mm × 80 mm. The non-motorised module has 4 wheels of Ø30 mm distributed in two pairs that press against the duct walls for stability. Overall dimensions are 75 mm × 62 mm × 74 mm. All three modules connect to the tensegrity by a hard transitional region and fulfilled pattern.

**Fig. 52:**
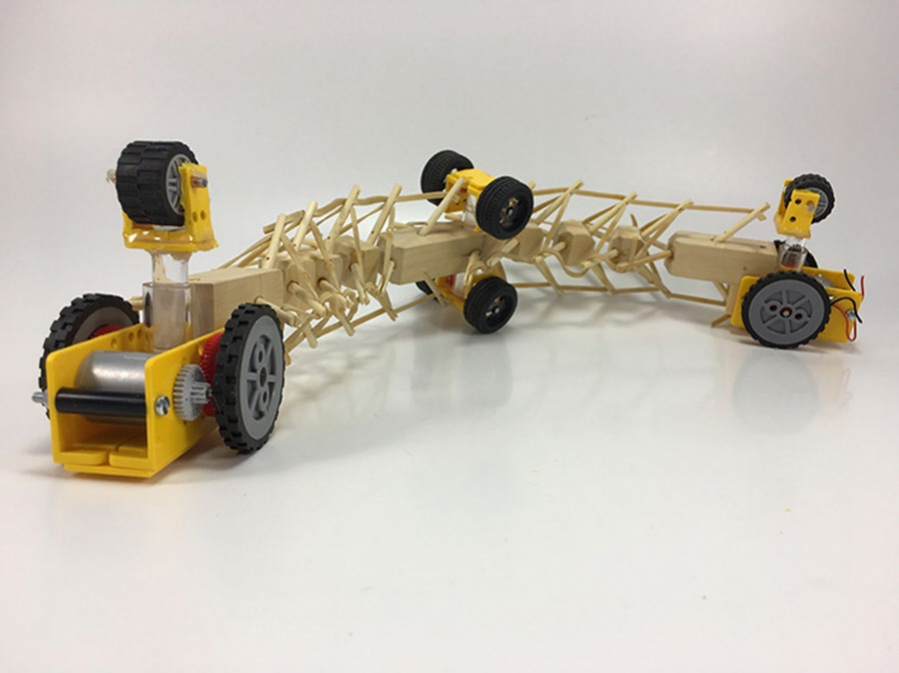
Wheeled tensegrity robot (WTR). It comprises three rigid structures and two tensegrity structures

**Fig. 53:**
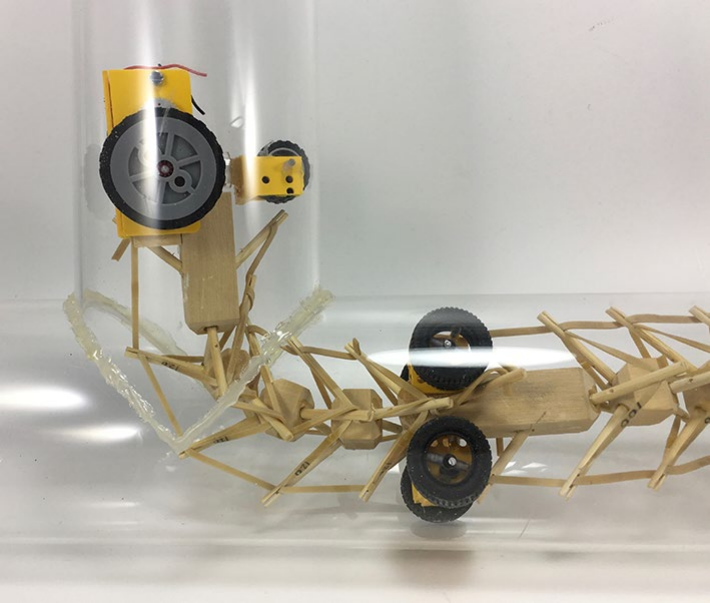
Wheeled tensegrity robot (WTR) turning inside a pipe

The robot has five sections. The first one is the front car, the second one is the front tetrahedral vertebral mast, the third one is the middle car, the fourth one is rear tetrahedral vertebral mast, and the fifth one is the rear car, as shown in Fig. 52. Overall dimensions are 500 mm × 62 mm × 80 mm. Total weight is 303 g. A pipe environment was built to assess the robot’s flexibility in operation. The model was capable of turning in horizontal and vertical planes inside the pipe as shown in Fig. 53. This kind of flexibility would require a high level of mechanical complexity in a conventional mobile robot. This robot was built in a very short amount of time with low cost of materials.

### Robot features

Having finalised the design and before testing the mechanical characteristics of the robot, we present a brief discussion regarding its features. They are introduced after setting up a frame of reference for this comparison.

Duct travelling robots can be classified in four categories: (1) conventional wheeled robots, (2) expanding robots, (3) tensegrity robots, and (4) hybrid tensegrity wheeled robots.

Conventional wheeled robots are the most common. Sometimes they use tracks to add more traction and climb ducts with a small slope. Expanding robots have arms that extend to reach for the walls of the ducting and press against them to gain the traction needed for vertical climbing; there are few of these robots in the market. Regarding tensegrity robots that travel in air ducts, to the authors’ knowledge there is only one created as part of a research project and it is DuCTT which is included in the literature review. The fourth category is what is presented in this paper: a hybrid tensegrity structure and solid-body wheeled robot that we refer to as a wheeled tensegrity robot (WTR).

This comparison is based on eight key mobility features that an air duct travelling robot should ideally have: (1) move horizontally inside a duct, (2) move vertically inside a duct, (3) turn right or left into a new branch (as opposed to just follow through a bend in the duct), (4) turn up or down into a new branch, (5) horizontal u-turn into a parallel adjacent branch with a narrow radius (6) vertical u-turn into a parallel adjacent branch in a narrow radius, (7) travel at standard speed for commercially available mobile robots and (8) move in an open plane, when not inside a duct.

Table 1 shows a summary of this comparison. Here, it can be clearly seen that some robots have some features, but none of them with the exception of a WTMR have all features available in one design.

**Table 1:** Comparison of four categories of duct travelling robots with respect to desired mobility features

	WTR	DuCTT	Wheeled robots	Expanding robots
Move horizontally	✔	✔	✔	✔
Move vertically	✔	✔	✗	✔
Turn right/left into a branch	✔	✔	✔	✗
Turn up/down into a branch	✔	✔	✗	✗
Narrow horizontal u-turn	✔	✗	✔	✗
Narrow vertical u-turn	✔	✗	✗	✗
Speed of 0.7 m/s or higher	✔	✗	✔	✔
Move in an open plane	✔	✗	✔	✔

**Fig. 54:**
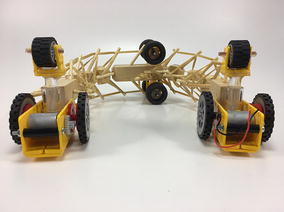
WTMR performing a u-turn with a small radius

**Fig. 55:**
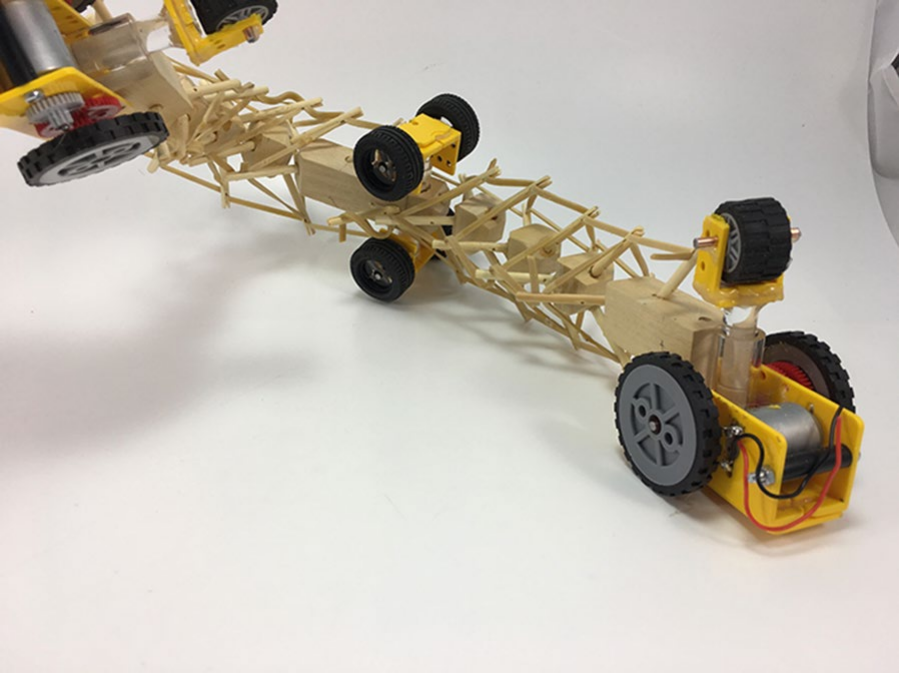
WTMR turning into a complex angle and twisting its body simultaneously

This table provides a basic example of the mobility that a WTR is capable of. The compliant structure can provide many other capabilities, but this brief list is indicative of the robot’s mobility within ducts. With appropriate actuation, a WTR can turn into branches of any direction, not just vertical or horizontal, and also twist its body in the axial direction simultaneously. Figures 54 and 55 show how the robot is capable of u-turns and turning in complex directions with multiple simultaneous rotations.

## Physical testing and discussion

### Rubber characterisation

**Fig. 56:**
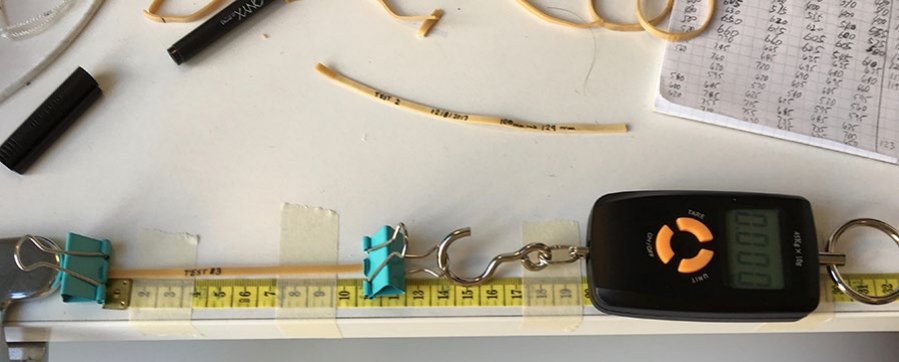
Rubber band test setup

To better understand the performance of the prototype, and by extension, future generations of similar WTMRs, three tests were performed to analyse the mechanical characteristics of the rubber band used to build the prototypes. In all three, 100 mm rubber band samples were used. Figure 56 shows how the test was set up. One end of the rubber band was fixed, while the other end was attached to a digital scale.

**Fig. 57:**
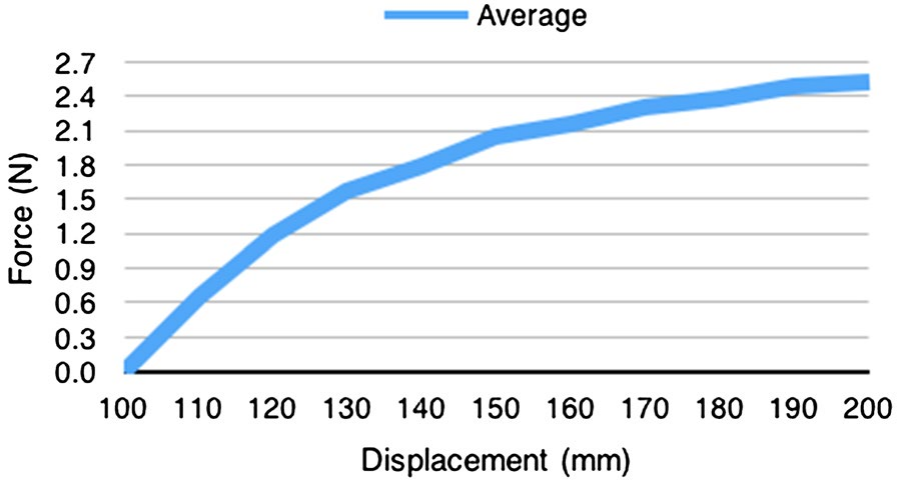
Rubber band test 3: force versus displacement. Test of 100 mm rubber band. Average of 15 times per displacement. Elastic zone determined was within 120 mm

The results showed that the mechanical properties of the rubber band deteriorated after a 20% displacement. Therefore, in order to have consistent results any deformation further than 20% was avoided. There was no noticeable linear pattern in the 20% deformation region; therefore, a simple linear formula was avoided and the tension in the cable was determined from interpolation of the experimental results obtained in Fig. 57.

### Tensegrity spine

**Fig. 58:**
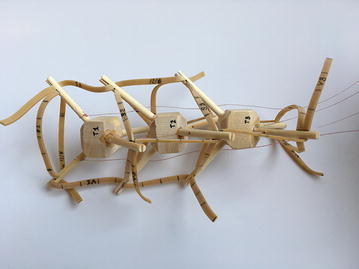
Unloaded test sample

**Fig. 59:**
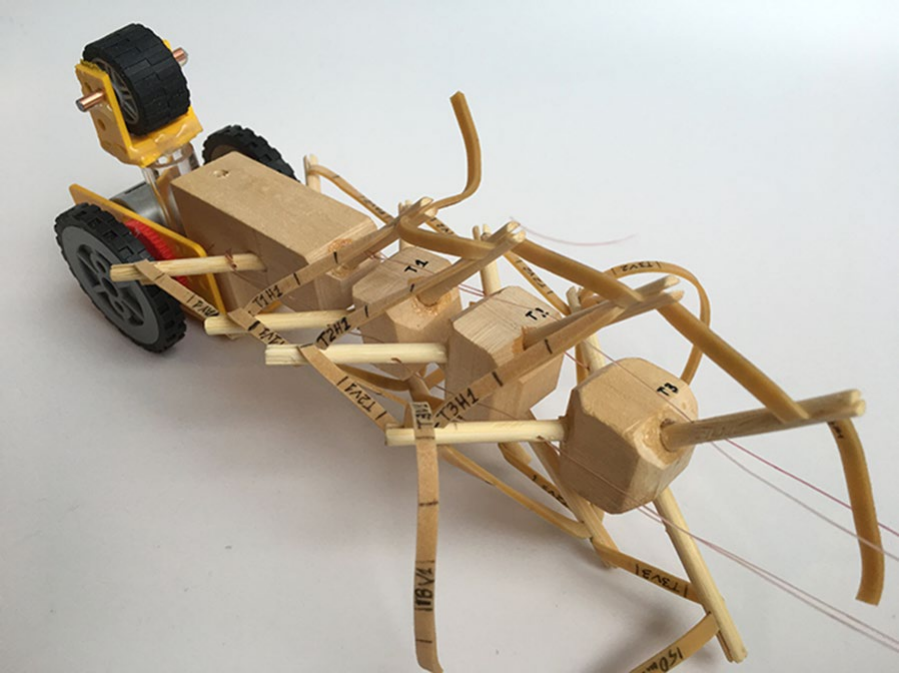
Loaded test sample

The first set of tests was performed on three stages of the tensegrity spine (T1, T2, T3). The purpose of the tests was to find the tension supported by each cable and how much tension was needed to bend the spine when unloaded and when linked to a load. The tensegrity spine was made out of soft wood and bamboo sticks. Rubber bands were used as cables. Custom-made rubber band loops were made with specific lengths (gluing together the ends) to be used as horizontal cables. The actuator was simulated with fishing nylon thread attached on the one end to the first strut in the structure and on the other end to a digital scale. Figure 58 shows the unloaded test sample, and Fig. 59 shows the loaded test sample with the front car as load.

**Fig. 60:**
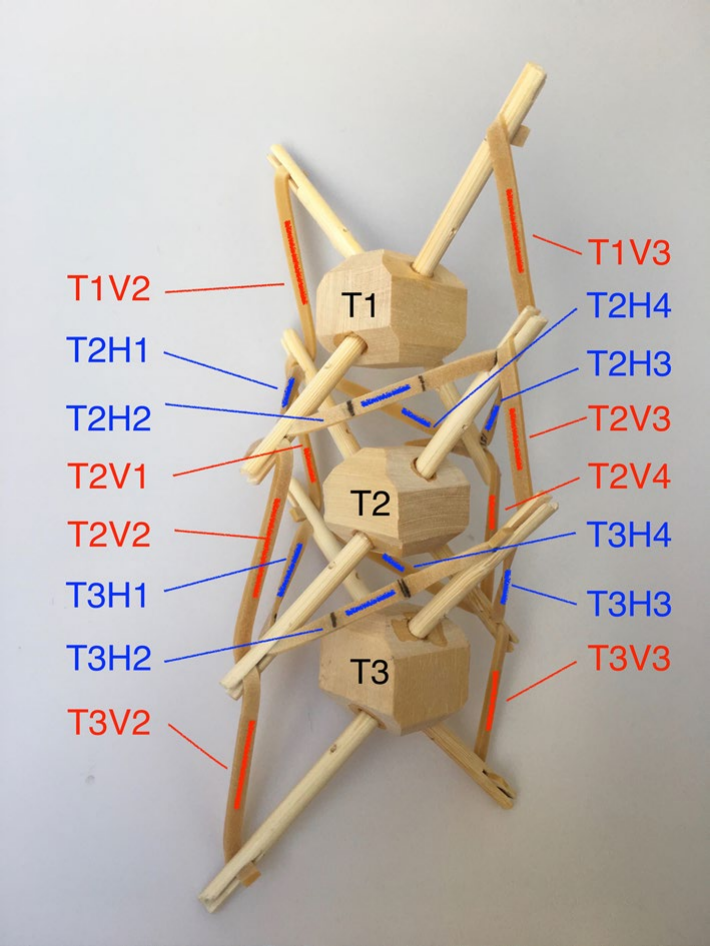
Labels for each cable in the testing prototype

A labelling system of two letters and two digits was used to identify each cable in this structure as shown in Fig. 60. The first half of the label belongs to the stage where the cable belongs: T1, T2, and T3. The second half of the label describes whether the cable is horizontal (tensional element of expansion, see tetrahedral vertebral mast) or vertical (tensional element of contraction). In Fig. 60, vertical cables are coloured red and horizontal cables are coloured blue for clarity.

The test consisted on measuring final length of 10 mm marks made on each cable. By measuring the displacement, the tension on the cables could be approximated. Tension in the actuator was measured with a digital scale.

#### *Test 1*

Rubber band loops made were 120 mm long which means that each horizontal cable was 30 mm long. Each vertical cable was also 30 mm long. Displacement was measured with no actuation and no loads acting upon the structure. Cable T3H4 (horizontal cable on the back left of rigid element T3) was the most loaded, showing a displacement of 70%. It should be noted that this cable shows more tension due to the fact that the rigid elements are not perfectly symmetric. In later tests, this was compensated by slightly varying the length of the cables. Table 2 shows the percentage displacement in all rubber band cables. Such high displacements over 20% greatly reduce the consistency of the data obtained. Therefore, the length of all horizontal cables had to be increased. Table 3 shows the calculation of the final length of rubber band loop T3H that comprise cables T3H1, 2, 3, and 4. The next test required a new set of rubber band loops that work under 20% displacement.

**Table 2:** Tensegrity spine test 1

T2H1	30%	T3H1	35%	T1V1	10%	T2V1	20%
T2H2	40%	T3H2	50%	T1V2	10%	T2V2	15%
T2H3	30%	T3H3	50%	T3V3	15%	T2V3	10%
T2H4	45%	T3H4	70%	T3V4	15%	T2V4	20%

Percentage of displacement in each horizontal (H) and vertical (V) cables

**Table 3:** Final length of rubber band loop T3H (mm). Calculated by adding displacements of T3H1, T3H2, T3H3, T3H4

	Disp. (%)	Initial length	Final length
T3H1	35	30	40.5
T3H2	50	30	45
T3H3	70	30	51
T3H4	50	30	45
Total			182

#### *Test 2*

For this test, 152 mm long rubber bands were prepared. With a displacement of 20%, the final length of the band was 182 mm which covered the displacement seen in Test 1. Test 2 comprises a set of six different runs. The first two runs were done with no actuation nor load. Runs 3 to 6 have an actuator cable to bend the tensegrity spine up, down, left, and right. The tension in this cable was measured as well. Run 1 showed cable T3H3 with a displacement of 25%. The length of the cable was slightly adjusted before the second run. Run 2 shows that this displacement was reduced to less than 20%; right under operational range. The next runs were under actuation to bend the tensegrity spine in four different directions. Two major displacements have been highlighted in Table 4. From these two, run 4 shows the most extreme results. Vertical cables are within limits. The measurements and calculations to get the final length of rubber band loop T3H are shown in Table 2. Total length of T3H was calculated to be 193 mm. Therefore, a new length for the loops needed to be used.

**Table 4:** Tensegrity spine test 2

	1	2	3	4	5	6
			Up	Down	Left	Right
T2H1	10%	10%	20%	10%	20%	10%
T2H2	10%	10%	25%	15%	10%	20%
T2H3	10%	10%	8%	20%	0%	30%
T2H4	10%	10%	30%	30%	*40%*	20%
T3H1	10%	10%	30%	20%	25%	10%
T3H2	19%	15%	30%	20%	20%	30%
T3H3	25%	18%	20%	*40%*	10%	35%
T3H4	15%	18%	20%	30%	30%	10%
T1V1	5%	5%	0%	0%	0%	10%
T1V2	0%	0%	0%	0%	10%	0%
T2V1	20%	20%	10%	0%	0%	10%
T2V2	10%	10%	0%	0%	10%	0%
T2V3	10%	10%	0%	10%	0%	0%
T2V4	11%	11%	20%	0%	10%	5%
T3V3	10%	10%	0%	10%	0%	0%
T3V4	9%	9%	10%	0%	0%	0%
T	0	0	1.9	1.7	2.1	2.1

Runs 1 and 2 have no actuation. Runs 3–6 have a cable actuator in order to bend the tensegrity spine up, down, left, and right. Tension of actuator T(N)

Displacement of rubber shown as percentage of original length

#### *Test 3*

Rubber band loops of 160 mm were prepared for the third test. With a displacement of 20%, a final length of 192 mm will be covered. The first 2 runs were made with no load and no actuation just to compensate for any peaks in tension in any of the cables. Indeed, some of the cables needed adjustment like T2H4 and T3H4 that were adjusted from 16 to 6 and 13 to 8%, respectively. Runs 3–6 were performed with actuation and no load. All displacements were under 20% this time. Runs 7–10 were performed with the front car (94 g) attached to the tensegrity spine. The results in Table 5 show a maximum displacement of 27% on cable T3H4 and a maximum actuation force of 12.4 N.

**Table 5:** Tension in horizontal cables (N)

	3	4	5	6
T2H1	0.96	0.06	0.9	0.42
T2H2	1.2	0.3	0.48	1.2
T2H3	0.48	1.2	0.48	1.2
T2H4	0.78	0.72	0.72	0.72
T3H1	1.02	0.36	1.02	0.36
T3H2	1.08	0.54	0.66	1.2
T3H3	0.78	0.78	0.6	1.2
T3H4	0.78	0.96	1.08	0.66

Interpolation of tension based on displacement of rubber

The use of rubber bands as a tension medium proved to be a limiting factor since the beginning of the experiments due to the quick degradation of its mechanical properties. Nevertheless, it was a quick and cheap method of prototyping and some useful information could still be drawn from the tests. It was determined that the working zone for this rubber band was under 20% displacement and 1.2 N of force. With these conditions, it was determined in Test 2 an optimal length of 160 mm for horizontal rubber band loops and 30 mm for vertical rubber bands. However, Test 3 demonstrated that the front car, weighing 94 g, was too big a load for the rubber bands. Additionally, it was noticed that in the runs with the 94 g load the tensegrity rigid elements started touching each other. This meant the tensegrity stopped behaving as such; cables in the tensegrity structure did not bear all loads anymore. The structure was overwhelmed by the load. Runs 3–6 in Test 3 can be used as an approximated method to calculate the loads on each cable based on the tension in the actuator, displacement, and Fig. 58.

## Conclusions

We have detailed how the very first wheeled tensegrity robot was successfully designed and prototyped. It was proven that this robot addressed all design requirements for a duct traveller mobile robot. This novel design proved to have capabilities not found in just one robot. There are robot designs for vertical climbing, designs for facilitating turns to the right and left, and designs that focus on speed and efficiency of mobility, but none of them group all these requirements into a single structure like the wheeled tensegrity robot does.

This research furthers the knowledge in the ever-growing field of tensegrity mobile robotics. It actually opens a new sub-category of hybrid wheeled tensegrity robotics. The project methodology was proven successful as it led to an optimised design that fulfilled all requirements. It had an advantage over simulation and analytical methods in these particular circumstances. It certainly was best to approach the research by broadly study structures and build models to assess their properties. The criteria of evaluation were validated by prototyping. Accepted designs by the criteria were stable. Rejected designs by the criteria were unstable. Furthermore, these criteria need to be expanded. The author sees many more possibilities of growth that were not pursued due to time constraints. Tensegrity robots do not need to address all design requirements with a tensegrity solution. Hybrid approaches have just been proved to be valid. Practical applications should start to appear more in research work. Transition areas should not be constrained to mobile robotics. In civil engineering, they can be applied to add tensegrity structures to fixed structures.

Future work should include testing the locomotive efficiency of this design. The weight can be further lowered by reducing the number of cars from three to only two and adding a tail section at the end for stability. It could also include computer simulation to refine the topology and morphology created, building a prototype with metal springs and a metal body (possibly aluminium) and adding motorised actuators to control the structure. Flexinol was also tested on this prototype as actuator, but the amount of actuation was much smaller that the structure required. However, a pulley arrangement is a possibility to be tested. Future work could also include exploring other topologies applied to wheeled tensegrity robotics and continue developing the criteria and designs for soft transitional regions.
